# Comorbid Depressive and Anxiety Symptoms and Their Correlates Among 93,078 Multiethnic Adults in Southwest China

**DOI:** 10.3389/fpubh.2021.783687

**Published:** 2021-12-14

**Authors:** Qiaolan Liu, Pingcuo Wangqing, Yangji Baima, Songmei Wang, Zhuozhi Shen, Jing Zhou, Huan Song, Yuanyuan Liu, Xiang Liu, Peng Luo, Xing Zhao

**Affiliations:** ^1^West China School of Public Health and West China Fourth Hospital, Sichuan University, Chengdu, China; ^2^Tibet Center for Disease Control and Prevention, Lhasa, China; ^3^Department of Preventive Medicine, College of Medicine, Tibet University, Lhasa, China; ^4^School of Public Health, Kunming Medical University, Kunming, China; ^5^Chongqing Municipal Center for Disease Control and Prevention, Chongqing, China; ^6^Chenghua District Center for Disease Control and Prevention, Chenghua, China; ^7^West China Biomedical Big Data Center of West China Hospital, Medical Big Data Center, Sichuan University, Chengdu, China; ^8^The Key Laboratory of Environmental Pollution Monitoring and Disease Control, School of Public Health, Ministry of Education, Guizhou Medical University, Guiyang, China

**Keywords:** comorbid depressive and anxiety symptoms, multiethnic adult, chronic disease, physical and mental well-being, psychosocial factor

## Abstract

**Background:** Depressive symptoms and anxiety symptoms commonly coexist and severely increases the disease burden worldwide. Little is known about the patterns and correlates of comorbid depressive and anxiety symptoms among the multiethnic populations of China.

**Methods:** This population-based study investigated the comprehensive associations of comorbid depressive and anxiety symptoms with lifestyles, stressful life events, chronic diseases, and physical and mental well-being among 93,078 participants (37,193 men, 55,885 women) aged 30–79 years across seven ethnic groups in Southwest China. Multivariable logistic regression models were used to estimate associations.

**Results:** Overall, 2.9% (2.1% in men and 3.5% in women) participants had comorbid depressive and anxiety symptoms; there was considerable heterogeneity among multiethnic populations. Participants with chronic diseases were more likely to have comorbidity than those without them; people with rheumatic heart disease reported the highest risk, with an odds ratio (OR) of 6.25 and 95% confidence interval (CI) of 4.06–9.62. Having experienced 3 or more stressful life events (OR, 8.43, 95% CI: 7.27–9.77), very poor self-rated health status (OR, 33.60, 95%CI: 25.16–44.87), and very unsatisfied life (OR, 33.30, 95% CI: 23.73–46.74) had strong positive associations with comorbid depressive symptoms and anxiety symptoms, with a dose-response relationship (*P* < 0.05). High frequency of physical activity had negative associations. All the associations were stronger than depressive symptoms alone or anxiety symptoms alone.

**Conclusions:** Our findings emphasize the need to focus on the vulnerable ethnic groups with comorbid depressive and anxiety symptoms, ultimate for help early prevention and improvement of health equity in the underdevelopment and high urbanization areas.

## Introduction

Depression and anxiety are two highly prevalent and disabling mental disorders that commonly coexist (1, 2). Comorbid depression and anxiety can occur in individuals of all ages, seriously impairing social function, increasing psychosocial disability, and reducing the quality of life. Moreover, comorbid depression and anxiety are more likely to increase recurrence and lead to suicide; it is associated with an even higher risk of mortality than depression alone or anxiety alone. Globally over 264 million people of all ages suffer from depressive disorder; it is one of the top three leading causes of years lived with disability (YLDs) for both sexes combined in the world (3). The prevalence estimate for anxiety disorders of 7.3% from a systematic review for 44 countries constitutes a huge global burden of disease (4). Mental and substance use disorders account for 7.4% of all disability-adjusted life years (DALYs) worldwide, in which depressive disorder account for 40.5% and anxiety disorders account for 14.6% (5). Identifying and managing comorbidities is essential because depression and anxiety, even if they are independent, contribute to a significant proportion of the global burden of disease (4, 6, 7).

Depressive symptoms and anxiety symptoms are reversible at early stages and can be treated with low-cost therapeutics (8, 9). Early interventions directly promote the treatment effects of other chronic diseases and reduce the burden of diseases, in turn leading to reduced health resource consumption (10). To achieve early management and intervention, early screening of comorbid depressive and anxiety symptoms warrants exploration.

Evidence on early screening of comorbid depressive and anxiety symptoms in mainland Chinese community populations is scarce. A limited number of studies are either based on elderly populations or in small sample community populations (11, 12), underscoring the importance of conducting studies in large general populations. Furthermore, research in multicultural vulnerable populations is limited, especially in China, where is economically underdeveloped yet has undergone rapid urbanization in recent years. Urbanizing leads to changes in the living environment and lifestyles, culture adjustment, job competition, life stress, and thus might cause mental health problems (13–15). The lack of scientific information on comorbid depressive and anxiety symptoms hinders policymakers from developing health services and allocating sufficient resources and intervention programs in these areas.

In this study, we examined the prevalence of comorbid depressive and anxiety symptoms among seven ethnic groups, including Tibetan, Miao, Bai, Bouyei, Dong, Yi, and Han in Southwest China using baseline data from the China multiethnic cohort (CMEC) (16). We also investigated the comprehensive factors that might influence the development of comorbid depressive and anxiety symptoms, including socioeconomic characteristics, lifestyles, stressful life events, sleep disorders, chronic diseases, and physical and mental well-being.

## Materials and Methods

### Study Participants

Participants included in this study were from the baseline survey of CMEC conducted between May 2018 and September 2019 in Southwest China. The design and conduct of CMEC have been described elsewhere (16). Briefly, the CMEC was established in five provinces of Southwest China, including Tibet, Sichuan, Chongqing, Guizhou, and Yunan. In addition to the Han ethnic group, six ethnic minority community populations (Tibetan, Yi, Bai, Miao, Dong, and Bouyei) were included in consideration of both sex ratio and age ratio using a multistage, stratified cluster sampling method. The inclusion criteria and exclusion criteria were strictly executed in the process of the CMEC study. Overall, 99,556 participants aged 30–79 years (18–79 years in Tibetan sample) and of the characteristics of permanent residents were enrolled in the CMEC, including 55,443 Han, 12,730 Tibetan, 6,283 Yi, 6,310 Bai, 5,559 Miao, 7,239 Dong, and 5,992 Bouyei. The current study included 93,078 participants aged 30–79 years (mean 51.8 years, SD 11.5 years) who were surveyed for both depressive symptoms and anxiety symptoms.

### Data Collection

A tablet computer with a self-developed application (CMES App) that had an automatic audio recording function was used to collect the questionnaire information by a face-to-face interview. The whole interview was implemented by well-trained interviewers who were medical workers from the local medical institutions or medical students from the local colleges, particularly familiar with the local ethnic language. The medical examinations provided information including height, weight, blood pressure, heart rate, ultrasound, X-rays, blood, and urine tests. Medical examinations and blood and urine collection strictly followed standard operating procedures (SOPs). The questionnaire covered socioeconomic status (age, gender, marital status, education, family income, occupation), lifestyle habits (smoking, alcohol drinking, tea consumption, dietary habits and physical activity), stressful life events, sleeping patterns, personal and family medical history, and psychological conditions.

### Assessment of Depressive Symptoms and Anxiety Symptoms

Depressive symptoms and anxiety symptoms were investigated using the PHQ-2 and GAD-2 questionnaires, respectively. These two questionnaires are two-item screening questionnaires based on the Diagnostic and Statistical Manual of Mental Disorders-Fourth Edition (DSM-IV) to assess depressive and anxiety symptoms in the past two weeks, respectively through the following two problems: (1) little interest or pleasure in doing things, and (2) feeling down, depressed, or hopeless (PHQ-2); (1) feeling nervous, anxiety, or on edge, and (2) not being able to stop or control worrying (GAD-2). For each item, the response options are “Not at all,” “Several days,” “More than half the days,” and “Nearly every day,” scored as 0, 1, 2, and 3, respectively. The total score of both the PHQ-2 and GAD-2 ranges from 0 to 6. A cutoff of 3 was adopted to identify depressive symptoms and anxiety symptoms. These two ultrashort screening tools considerably enhance the efficiency of screening for and monitoring depressive symptoms and anxiety symptoms in busy primary care practice and large population-based epidemic surveys (17–21).

We conducted a repeated study with a sample of 10% of the total population from the baseline study from June to October 2020. The questionnaire survey, medical examination, and biometric sample collection were mostly the same as the baseline, but the PHQ-9, GAD-7 and a few other questions were added. The 8,229 participants of Han, Yi, Bai, and Miao nationality from 10% of the repeated study population were surveyed simultaneously on four scales of PHQ-9, PHQ-2, GAD-7, and GAD-2. PHQ-9 and GAD-7 classify diagnoses according to the Diagnostic and Statistical Manual of Mental Disorders fourth edition (DSM-IV) (18, 22). The Cronbach alpha coefficients were 0.73 for the PHQ-2 and 0.74 for the GAD-2 in this study. The correlation between the PHQ-9 and the PHQ-2 was 0.813. The Cronbach's alpha coefficients for the PHQ-9 and PHQ-2 were 0.809 and 0.691, respectively. Using the PHQ-9 with a cutoff of 10 as the threshold value, the area under the ROC curve for the PHQ-2 was 0.948, and the specificity and sensitivity of the PHQ-2 were 98.2 and 64.6%, respectively. The correlation between the GAD-7 and the GAD-2 was 0.906 in this same sample. The Cronbach's alpha coefficients of the GAD-9 and GAD-2 were 0.898 and 0.749, respectively. Using GAD-7 with a cutoff of 10 as the threshold value, the area under the ROC curve for GAD-2 was 0.987, and the specificity and sensitivity of GAD-2 were 98.2 and 92.9%, respectively. All these results demonstrated good reliability and validity of the PHQ-2 and GAD-2 for our population sample.

### Assessment of Sleep Disorders

Participants were asked whether they had any of the following three symptoms in at least three days or more in a week in the past month: (1) having trouble falling asleep after going to bed or waking up in the middle of the night; (2) waking up too early and not being able to get back to sleep; and (3) having trouble staying alert while at work, eating or meeting people during the daytime. Participants who answered “Yes” to any of the above three items were classified as having sleep disorders (23).

### Assessment of Stressful Life Events

Participants were asked whether they had experienced the following 10 stressful events during the past two years: (1) divorce/separation; (2) loss of job/retirement; (3) business failure or bankruptcy; (4) being violently attacked/raped; (5) serious family internal contradictions and conflicts; (6) a serious injury or car accident; (7) seriously ill or death of a spouse; (8) seriously ill or death of other close family members; (9) serious natural disasters (e.g., drought or flood); (10) loss of the source of income/living in debt (24).

### Assessment of Life Satisfaction and Self-Rated Health Status

One item was designed to ask participants whether they were satisfied with their life. The answer was grouped into five orderly categories as “very satisfied,” “satisfied,” “general,” “not satisfied,” and “not satisfied at all.” Participants were also asked what they think of their current health status. The answer was also grouped into five orderly classes: “very good,” “good,” “medium,” “poor,” and “very poor” (24).

### Assessment of Common Chronic Diseases

Information about the occurrence of cancers and the top 20 kinds of common chronic diseases was collected from the local disease surveillance system in the Chinese western multiple minority areas. The diseases included rheumatic health disease, coronary heart disease, diabetes, stroke, and some plateau-specific diseases; these diseases must be confirmed by a medical institution at or above the level of the county hospital before the baseline survey.

### Statistical Analysis

The mean values and proportions with selected baseline characteristics were calculated by the presence of depressive symptoms, anxiety symptoms, and their comorbidity. To analyze changes of comorbidity of depressive and anxiety symptoms with age, we used the Chi-square trend test. To explore the potential influencing factors for depressive symptoms, anxiety symptoms, and their comorbidity, logistic regression models were used to calculate multivariable-adjusted ORs and 95% CIs. All the ORs in the tables and figures were adjusted simultaneously for age grouped into five categories per 10 years: gender, education (illiteracy, primary, middle school, and college), family income (<12,000, 12,000–19,999, 20,000–59,999, 60,000–99,999, 100,000–199,999, and ≥200,000 RMB/year), occupation (employed, retirement, unemployment), and marriage (married, divorced, widowed, and single). The other variables included smoking (no, past, current), alcohol consumption (no, past, current), tea consumption (no, yes), BMI (<18.5, 18.5–23.9, 24–27.9, and ≥28 kg/m^2^), and physical activity (<12.0, 12.0–37.3, ≥37.3 MET-h/day). The frequency of tea consumption was classified into four categories: past, 1–2, 3–5, and 6–7 d/week. The duration of tea consumption was grouped into three categories according to three quantiles: <15, 15–39, and ≥39 years. The frequency and duration of alcohol consumption and the duration of smoking were also grouped into three categories using the same methods. A trend test was conducted by entering the categorical variables as continuous parameters in the models. All analyses were conducted using R software, version 3.6.3 (R Core Team). The threshold for statistical significance was *P* ≤ 0.05 based on 2-sided tests.

## Results

**Figure 1:**
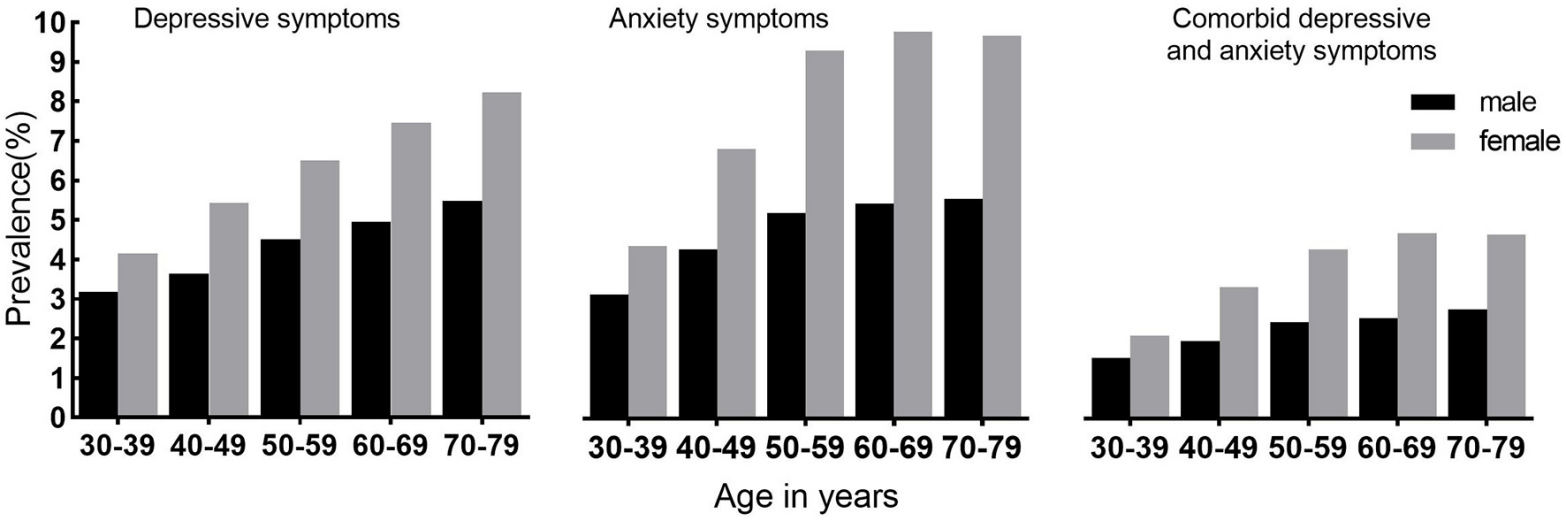
Prevalence of depressive symptoms and anxiety symptoms and comorbidity by age and sex.

Overall, 2.9% of participants reported having comorbid depressive and anxiety symptoms, 2.1% among men and 3.5% among women (Table 1). Among 4,947 (5.3%) participants with depressive symptoms, 55.0% reported having anxiety symptoms, while in 5,800 (6.2%) participants with anxiety symptoms, 46.9% reported having depressive symptoms. Those participants with depressive symptoms had a 27-fold elevated risk of anxiety symptoms compared with participants with no depressive symptoms (Supplemental Figure 1). For both men and women, the prevalence of comorbid depressive and anxiety symptoms increased until ~60 years (*P* trend <0.001); after 70 years, it was almost the same (Figure 1). Compared with participants who were neither depressive symptoms nor anxiety symptoms, those with comorbid depressive and anxiety symptoms were more likely to have poor education, lower household income, and be widowed/divorced (Table 1).

**Table 1:** Sociodemographic characteristics and selected characteristics by mental health status.

	**Overall**	**Depressive symptoms**	**Anxiety symptoms**	**Neither depression nor anxiety**	**Comorbid depressive and anxiety symptoms**
	**(*N* = 93,078)**	**(*n* = 4,947)**	**(*n* = 5,800)**	**(*n* = 85,052)**	**(*n* = 2,721)**
Age (*x* ± SD, years)	51.8 ± 11.5	53.9 ± 11.5	54.1 ± 11.0	51.6 ± 11.5	54.1 ± 11.1
**Age (years) (%)**					
30-	15,733	596 (3.8)	584 (3.7)	14,832	279 (1.8)
40-	28,265	1,341 (4.7)	1,574 (5.6)	26,098	748 (2.7)
50-	24,759	1,422 (5.7)	1,823 (7.4)	22,349	835 (3.4)
60-	17,515	1,113 (6.5)	1,314 (7.5)	15,704	616 (3.5)
70-79	6,806	475 (7.0)	505 (7.4)	6,069	243 (3.6)
**Gender (%)**					
Male	37,193	1,569 (4.2)	1,660 (4.5)	34,736	772 (2.1)
Female	55,885	3,378 (6.0)	4,140 (7.4)	50,316	1,949 (3.5)
**Education (%)**					
Illiteracy	23,277	1,937 (8.3)	2,458 (10.6)	20,079	1,197 (5.1)
Primary	23,948	1,332 (5.6)	1,560 (6.5)	21,788	732 (3.1)
Middle school	35,477	1,376 (3.9)	1,545 (4.4)	33,235	679 (1.9)
College	10,375	302 (2.9)	237 (2.3)	9,949	113 (1.1)
**Marriage (%)**					
Married	82,658	4,095 (4.9)	4,871 (5.9)	75,941	2,249 (2.7)
Divorce	3,613	270 (7.5)	273 (7.6)	3,217	147 (4.1)
Widowed	5,692	514 (9.0)	600 (10.5)	4,880	302 (5.3)
Single	1,114	68 (6.1)	56 (5.0)	1,013	23 (2.1)
**Occupation (%)**					
Employed	79,425	4,289 (5.4)	5,198 (6.5)	72,355	2,417 (3.0)
Retirement	9,434	348 (3.7)	338 (3.6)	8,888	140 (1.5)
Unemployment	4,142	308 (7.4)	262 (6.3)	3,734	162 (3.9)
**Family income (RMB/YEAR) (%)**					
<12,000	16,466	1,572 (9.6)	1,855 (11.3)	14,005	966 (5.9)
12,000–19,999	16,468	931 (5.7)	1,159 (7.0)	14,903	525 (3.2)
20,000–59,999	33,325	1,579 (4.7)	1,867 (5.6)	30,709	830 (2.5)
60,000–99,999	13,997	481 (3.4)	539 (3.9)	13,209	232 (1.7)
100,000–199,999	10,145	302 (3.0)	305 (3.0)	9,670	132 (1.3)
≥200,000	2,562	71 (2.8)	66 (2.6)	2,455	30 (1.2)
**Nationality (%)**					
Han	55,281	2,448 (4.4)	2,415 (4.4)	51,583	1,165 (2.1)
Dong	7,222	764 (10.6)	1,045 (14.5)	5,883	470 (6.5)
Bouyei	5,954	400 (6.7)	641 (10.8)	5,154	241 (4.1)
Yi	6,276	406 (6.5)	565 (9.0)	5,574	269 (4.3)
Miao	5,538	633 (11.4)	809 (14.6)	4,500	404 (7.3)
Bai	6,106	163 (2.7)	177 (2.9)	5,863	97 (1.6)
Tibetan	6,701	133 (2.0)	148 (2.2)	6,495	75 (1.1)

**Figure 2:**
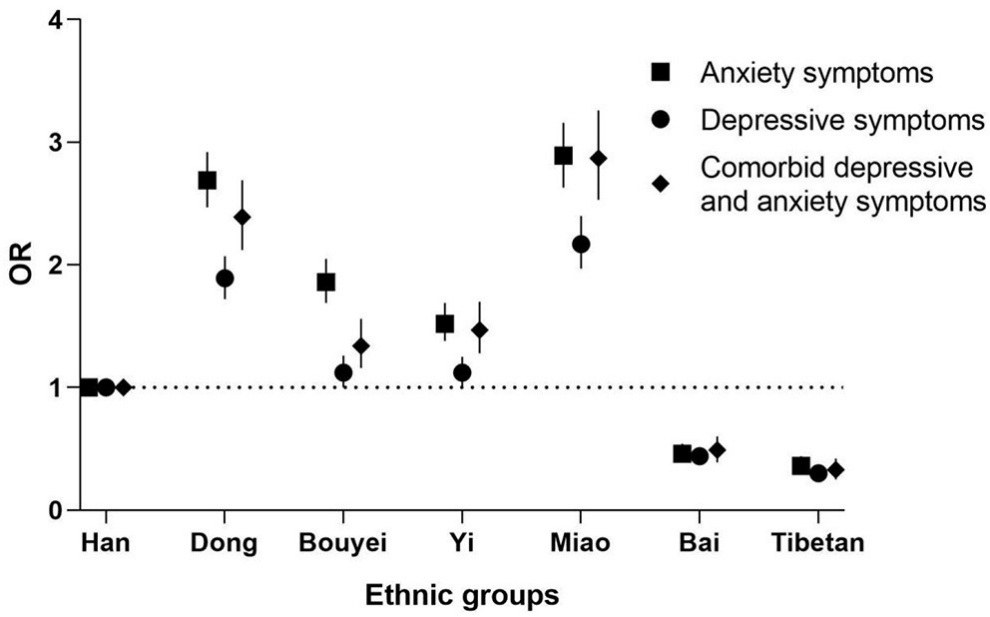
Odds ratios of depressive symptoms, anxiety symptoms, and comorbidity by minority groups compared with Han nationality. Each closed square, circle or rhombus represents an odds ratio, and the horizontal lines represent the 95% confidence interval (CI). All odds ratios were adjusted for age, sex, marriage, education level, occupation, family income, smoking, alcohol consumption, tea consumption, BMI, and physical activity.

Among the seven ethnic groups, Tibetans had the lowest level of comorbid depressive and anxiety symptoms at 1.1%. The Miao ethnic group had the highest levels of comorbidity (7.3%) (Table 1). Compared with the Han ethnic group in Figure 2, the Tibetan ethnic group had a lower risk of comorbidity (OR = 0.38, 95% CI: 0.29–0.50), and the low-risk result was also found among the Bai ethnic group (OR = 0.50, 95% CI: 0.40–0.63) after adjusting for age, sex, education, family income, and lifestyles. The Miao ethnic group had an increased risk of comorbidity (OR = 3.04, 95% CI: 2.65–3.48).

The associations between lifestyle factors and comorbid depressive and anxiety symptoms are presented in Table 2. Medium and high physical activity was inversely related to comorbidities, with ORs of 0.69 (95% CI: 0.62–0.76) and 0.82 (95% CI: 0.74–0.91), respectively. Frequent alcohol consumption was found to be inversely associated with comorbid depressive and anxiety symptoms; this was observed in most ethnic groups and men, but the opposite was found in women (Supplemental Table 1). We did not find that the frequency of tea consumption was associated with comorbid depressive and anxiety symptoms. We also did not find an association between smoking or BMI and comorbidity.

**Table 2:** Associations of lifestyles and behaviors with comorbid depressive and anxiety symptoms.

Variables^*^		**Depressive symptoms**		**Anxiety symptoms**		**Comorbid depressive and anxiety symptoms**	
	**Total (*N* = 93,078)**	**Yes (*n* = 4,947)**	**Odds ratio (95%CI)**	**Yes (*n* = 5,800)**	**Odds ratio (95%CI)**	**Yes (*n* = 2,721)**	**Odds ratio (95%CI)**
**Smoking**							
No	68,837	3,901	1	4,701	1	2,222	1
Past	4,700	213	1.11 (0.94, 1.30)	243	1.18 (1.01, 1.38)	113	1.15 (0.92, 1.43)
Current	19,541	833	1.00 (0.90, 1.11)	856	0.95 (0.86, 1.06)	386	0.87 (0.75, 1.01)
**Duration of smoking (years)**							
<24	5,934	232	1.12 (0.96, 1.31)	228	1.09 (0.93, 1.27)	107	1.03 (0.82, 1.29)
24–42	12,637	516	0.97 (0.86, 1.09)	586	1.01 (0.90, 1.14)	246	0.90 (0.76, 1.07)
≥42	5,610	294	1.04 (0.90, 1.21)	281	0.89 (0.76, 1.03)	144	0.87 (0.71, 1.07)
**Alcohol consumption**							
No	52,079	3,067	1	3,611	1	1,748	1
Sometime	28,411	1,380	0.94 (0.87, 1.01)	1,586	0.96 (0.90, 1.03)	732	0.92 (0.84, 1.01)
Frequently	12,588	500	0.76 (0.69, 0.85)	603	0.88 (0.80, 0.97)	241	0.71 (0.61, 0.82)
**Frequency of alcohol consumption**							
1–2 d/week	3,055	136	0.92 (0.77, 1.11)	159	1.04 (0.87, 1.24)	67	0.87 (0.67, 1.13)
3–5 d/week	2,557	89	0.69 (0.56, 0.87)	119	0.92 (0.75, 1.12)	45	0.69 (0.51, 0.94)
6–7 d/week	6,972	275	0.67 (0.59, 0.78)	324	0.78 (0.69, 0.89)	129	0.61 (0.50, 0.74)
**Tea consumption**							
No	63,106	3,774	1	4,594	1	2,135	1
Yes	29,969	1,172	0.98 (0.90, 1.05)	1,206	0.92 (0.85, 0.99)	586	0.95 (0.85, 1.05)
**Frequency of tea consumption**							
1–2 d/week	3,096	140	0.85 (0.71, 1.02)	144	0.96 (0.81, 1.13)	68	0.96 (0.75, 1.24)
3-5 d/week	3,862	131	0.98 (0.90, 1.06)	149	0.95 (0.80, 1.12)	64	0.87 (0.68, 1.13)
6–7 d/week	22,251	854	1.03 (0.86, 1.22)	858	0.88 (0.81, 0.96)	426	0.93 (0.83, 1.05)
**Duration of tea consumption (years)**							
<15	7,061	343	1.09 (0.97, 1.22)	351	1.00 (0.89, 1.12)	174	1.07 (0.91, 1.25)
15–39	15,513	564	0.94 (0.85, 1.04)	586	0.92 (0.83, 1.01)	280	0.92 (0.80, 1.05)
≥39	7,247	252	0.87 (0.75, 1.00)	254	0.76 (0.66, 0.88)	124	0.78 (0.64, 0.96)
**BMI**							
<18.5	2,954	211	1.18 (1.01, 1.37)	222	1.04 (0.89, 1.20)	114	1.13 (0.92, 1.38)
18.5–23.9	41,127	2,226	1	2,553	1	1,214	1
24–27.9	33,717	1,618	0.93 (0.87, 0.99)	1,929	0.97 (0.91, 1.03)	871	0.92 (0.84, 1.01)
≥28	11,334	629	1.01 (0.92, 1.11)	794	1.11 (1.02, 1.21)	353	1.04 (0.92, 1.17)
**Physical activity (MET-h/week)**							
<12.0	23,106	1,453	1	1,495	1	797	1
12.0–37.3	46,397	2,111	0.72 (0.66, 0.77)	2,526	0.85 (0.79, 0.91)	1,124	0.69 (0.62, 0.76)
≥37.3	23,216	1,355	0.82 (0.76, 0.89)	1,740	1.02 (0.95, 1.11)	784	0.82 (0.74, 0.91)

* *Adjusted for age, sex, nationality, marriage, education level, occupation, and family income*.

Table 3 shows the adjusted ORs for comorbid depressive and anxiety symptoms associated with 20 common chronic diseases by adjusting for sociodemographic characteristics and lifestyle habits. We estimated the associations of depressive symptoms, anxiety symptoms and comorbidities with each of these diseases separately. Individuals who reported having rheumatic heart disease were more likely to have comorbid depressive and anxiety symptoms with OR 6.25 (95%CI: 4.06–9.62); the other diseases also strongly related with the comorbidity were pulmonary heart disease with OR 3.56 (95%CI: 2.58–4.90), coronary heart disease with OR 2.94 (95%CI: 2.48–3.48) and stroke with OR 2.78 (95%CI: 2.04–3.79).

**Table 3:** The adjusted ORs of chronic diseases and comorbid depressive and anxiety symptoms.

Type of disease^*^		**Depressive symptoms**		**Anxiety symptoms**		**Comorbid depressive and anxiety symptoms**	
	**Total** **(*N* = 93,078)**	**With disease** **(*n* = 4,947)**	**Odds ratio** **(95% CI)**	**With disease** **(*n* = 5,800)**	**Odds ratio** **(95% CI)**	**With disease** **(*n* = 2,721)**	**Odds ratio** **(95% CI)**
**Diseases**							
No	38,517	1,412	1	1,757	1	760	1
yes	54,560	3,541	1.92 (1.8, 2.05)	4,049	1.78 (1.67, 1.89)	1,961	2.04 (1.87, 2.23)
Hypertension	16,063	1,124	1.34 (1.25, 1.45)	1,293	1.27 (1.18, 1.37)	632	1.38 (1.25, 1.52)
Diabetes	4,503	347	1.54 (1.37, 1.73)	373	1.47 (1.31, 1.66)	191	1.65 (1.41, 1.93)
Hyperlipidemia	7,366	468	1.72 (1.55, 1.91)	498	1.68 (1.52, 1.86)	259	1.93 (1.68, 2.22)
Coronary heart disease	2,478	309	2.57 (2.25, 2.92)	342	2.52 (2.22, 2.86)	178	2.94 (2.48, 3.48)
Stroke	675	80	2.41 (1.89, 3.08)	81	2.20 (1.72, 2.81)	48	2.78 (2.04, 3.79)
Rheumatic heart disease	212	40	4.70 (3.28, 6.75)	39	4.08 (2.83, 5.87)	27	6.25 (4.06, 9.62)
Pulmonary heart disease	398	80	3.21 (2.47, 4.17)	79	2.48 (1.90, 3.24)	51	3.56 (2.58, 4.90)
Tuberculosis	1,478	121	1.58 (1.30, 1.92)	138	1.67 (1.38, 2.01)	74	1.84 (1.44, 2.36)
Chronic bronchitis	5,794	568	1.93 (1.75, 2.12)	618	1.80 (1.64, 1.98)	328	2.12 (1.87, 2.40)
Asthma	1,320	154	2.29 (1.92, 2.73)	154	2.06 (1.72, 2.46)	81	2.37 (1.87, 3.01)
Hepatitis	2,589	200	1.95 (1.67, 2.27)	190	1.72 (1.47, 2.01)	99	1.94 (1.57, 2.39)
Peptic ulcer	2,699	232	1.98 (1.72, 2.28)	247	1.92 (1.67, 2.21)	136	2.33 (1.94, 2.79)
Gastroenteritis	11,687	974	1.94 (1.80, 2.09)	1,049	1.79 (1.66, 1.92)	563	2.15 (1.95, 2.37)
Gallstone	11,130	700	1.36 (1.25, 1.49)	786	1.31 (1.21, 1.42)	394	1.43 (1.28, 1.61)
Fracture	6,900	463	1.45 (1.31, 1.61)	528	1.50 (1.36, 1.65)	248	1.51 (1.32, 1.74)
Rheumatic arthritis	6,589	608	1.76 (1.60, 1.93)	728	1.80 (1.65, 1.96)	379	2.04 (1.81, 2.30)
Rheumatoid arthritis	2,556	281	2.24 (1.96, 2.56)	347	2.41 (2.13, 2.72)	186	2.80 (2.38, 3.30)
Intervertebral disc disease	16,710	1,343	1.68 (1.57, 1.80)	1,578	1.67 (1.57, 1.78)	785	1.87 (1.71, 2.04)
Cerebral trauma	1,900	183	1.94 (1.66, 2.28)	212	2.03 (1.74, 2.36)	99	2.10 (1.69, 2.60)
Cancer	780	66	1.54 (1.19, 1.99)	68	1.33 (1.02, 1.72)	38	1.62 (1.16, 2.27)

* *Adjusted for age, sex, nationality, marriage, education level, occupation, family income, smoking, alcohol consumption, tea consumption, BMI, and physical activity*.

**Figure 3:**
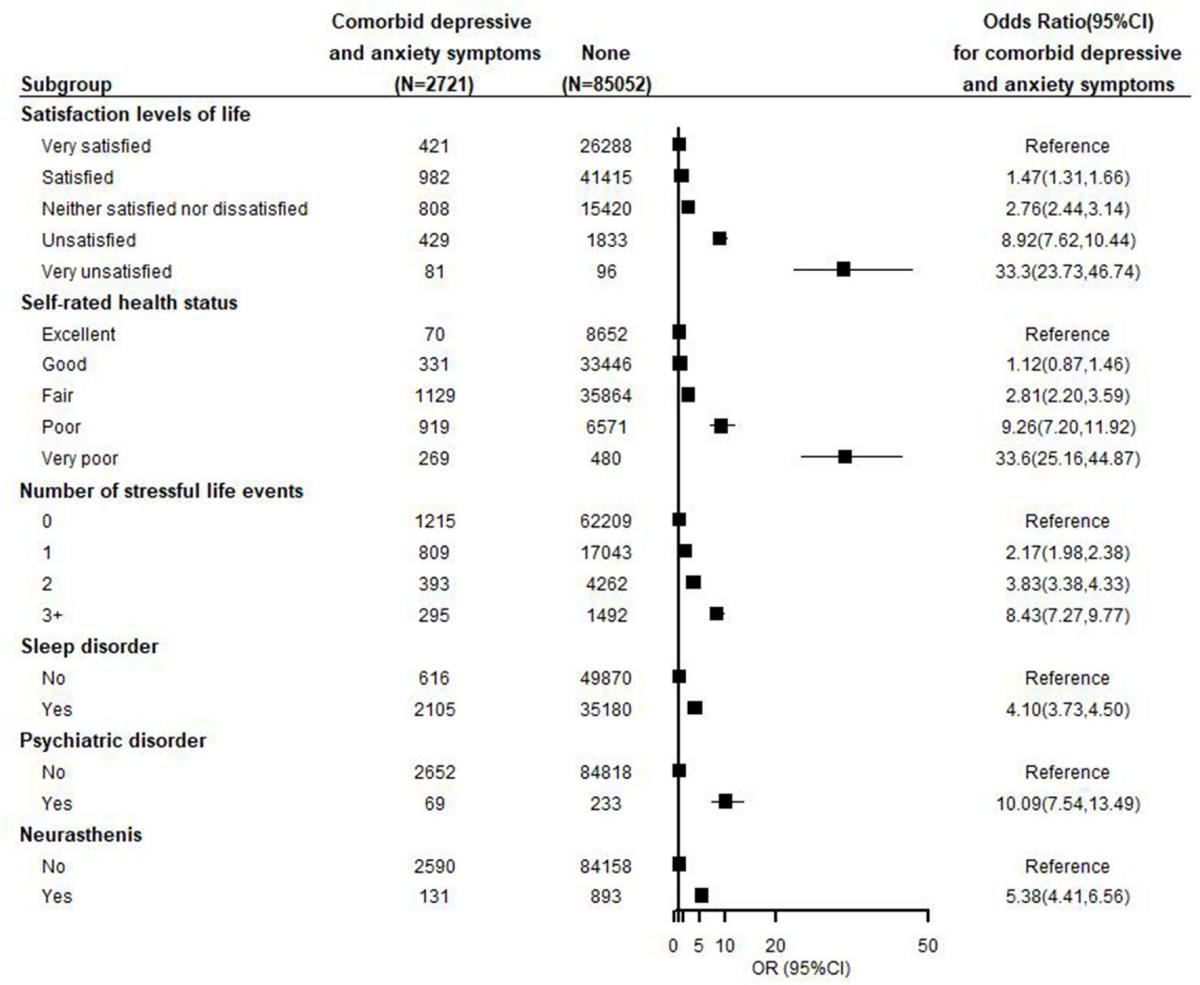
Adjusted odds ratios by health-related conditions and status for comorbid depressive and anxiety symptoms. Each closed square represents an odds ratio, and the horizontal lines represent the 95% confidence interval (CI). All odds ratios were adjusted for age, sex, nationality, marriage, education level, occupation, income, smoking, alcohol consumption, tea consumption, BMI, physical activity, and diseases.

Compared with those who were excellent self-rated health status, the adjusted ORs of comorbid depressive and anxiety symptoms were increased with the reduced degree of self-rated health status, the ORs of the comorbidity were 2.81(95%CI: 2.20–3.59) for “fair,” 9.26 (95%CI: 7.20–11.92) for “poor”, and 33.60 (95%CI: 25.16–44.87) for “very poor,” with dose-response relationship (trend *P* < 0.001). Similarly, compared with those with a very satisfying life, the adjusted ORs of comorbid depressive and anxiety symptoms increased with a reduced degree of self-reported life satisfaction, with ORs of 2.76 (95% CI: 2.44–3.14), 8.92 (95% CI: 7.62–10.44), and 33.30 (95% CI: 23.73–46.74) for “neither satisfied nor dissatisfied,” “unsatisfied,” and “very unsatisfied,” respectively, with a dose-response relationship (trend *P* < 0.001) (Figure 3).

Individuals who reported having sleep disorders, a history of doctor-diagnosed psychiatric disorder or neurasthenia had a higher likelihood of having comorbid depressive and anxiety symptoms; the ORs of these mental disorders associated with comorbidities were 4.10 (95% CI: 3.73–4.50), 10.09 (95% CI: 7.54–13.49), and 5.38 (95% CI: 4.41–6.56), respectively. The adjusted ORs increase progressively with the number of stressful life events experienced during the previous two years, with a dose-response relationship (trend *P* < 0.001) (Figure 3).

Moreover, the associations between all of the above factors and comorbid depressive and anxiety symptoms were higher than that of depressive symptoms alone or anxiety symptoms alone (Table 3; Supplemental Figures 1, 2).

## Discussion

In this large multiethnic population-based study with seven ethnic groups aged 30–79 years from 5 provinces in Southwest China, 2.9% of participants reported having comorbid depressive and anxiety symptoms. Comparing the prevalence of 4.2–12% of comorbid depressive and anxiety symptoms with a few previous studies, the total prevalence in our study was low (25, 26). The low prevalence could be underestimated because of the lack of mental health literacy and stigma-induced conscious non-disclosure reporting in the low-middle developing areas.

In the study, higher risks of comorbid depressive and anxiety symptoms were associated with female sex and low socioeconomic status, including poorer education, lower household income, and being widowed; the correlates were similar to depressive symptoms alone or anxiety symptoms alone. Surprisingly, frequent alcohol consumption was associated with lower risks of comorbid depressive and anxiety symptoms. These results were somewhat consistent with previous studies which demonstrated that moderate not heavy alcohol consumption had lower risk to have depression (27, 28). In this study, the rate of frequently drinking alcohol was 13.5% in total, 29.5% among men and 2.9% among women. A high frequency of drinking was almost 10 times more common in men than in women (Supplementary Table 1). Drinking alcohol, generally regarded as moderate drinking, is often combined with local culture and used to offer sacrifices and entertain guests in some ethnic groups. Drinking with other people is part of daily life entertainment among men from some ethnic groups. Therefore, it is not the alcohol itself but the activities of drinking, which might enable men to participate in social life and obtain social support, thereby reducing depressive symptoms and anxiety symptoms. Further gender-specific, the amount of alcohol consumption studies is needed to explore the associations of alcohol consumption with comorbid depressive and anxiety symptoms.

The Miao minority and Dong minority reported the highest risks of comorbid depressive and anxiety symptoms, followed by the Bouyei and Yi minorities. These ethnic minorities live in economically underdeveloped areas and were in the process of accelerating urbanization, which resulted in not only geographical environmental changes but also living modes, interpersonal communication modes, traditional values, and cultural changes (29, 30). Previous studies have shown that people from these urbanizing minority areas suffer from residential migration, lifestyle changes, and cultural adjustment and integration; compared with people already living in well-developed cities, these residents were more likely to have depression or anxiety (13, 14). The considerable differences in comorbid depressive and anxiety symptoms among ethnic groups could not only be explained entirely due to changes in the environment. There might also be differences in the interaction between genes and the environment, which requires more in-depth research (31, 32).

In this study, we investigated the top 20 common chronic diseases and cancers in the southwest multiethnic populations. We found that all chronic diseases and cancers were associated with comorbid depressive and anxiety symptoms; the associations were stronger than depressive symptoms alone or anxiety symptoms alone. Some previous studies have demonstrated that stroke, coronary heart disease, and cancers are strongly associated with depressive symptoms or anxious symptoms (33–36). We confirmed these results. Moreover, we found rheumatic heart disease had the highest risk with comorbid depressive and anxiety symptoms among these 20 kinds of diseases. Comorbid depressive sand anxiety symptoms can increase patients' medical overuse and are associated with slower recovery, reducing adherence to treatment than depressive symptoms or anxiety symptoms alone (37). These results suggested that, based on the current situation of the severe shortage of mental health resources in southwest minority areas, policymakers should formulate policies to improve comorbid depressive and anxiety symptoms among patients with key chronic diseases in basic community health centers or rural township health centers.

Although a simple question was designed to measure life satisfaction and self-rated health status, we found strong linear associations. Compared with those who were very satisfied with life and excellent health status, participants who were very unsatisfied with life and very poor health status had 33-fold and 34-fold higher risks of comorbid depressive and anxiety symptoms, respectively. This suggests that primary health care institutions should pay special attention to residents who complain about their life status or physical conditions because they are likely to suffer from depressive symptoms, anxiety symptoms or both (9).

Stressful life events as strong risk factors for depressive symptoms and anxiety symptoms have been reported in populations of different ages. One psychological mechanism suggests that rumination mediates the longitudinal relationship between stressful life events and comorbid depressive and anxiety symptoms (38), while the genetic mechanism suggests that stressful life events may change mitochondrial DNA and telomere length to cause depression (39). Previous studies have demonstrated that depressive symptoms and anxiety symptoms are associated with sleep disorders, other psychiatric disorders and neurasthenia (24, 40). The present study showed that the associations between these mental problems and the comorbidity were stronger than those of depressive symptoms alone or anxiety symptoms alone. Due to culture, customs and discrimination against mental disorders, many ethnic minority residents may report neurasthenia or sleep disorders if they suffer from depressive symptoms or anxiety symptoms (7, 41). The results suggested that general medical practitioners from primary care institutions should be given adequate training to improve the ability to recognize psychological problems to better identify and manage depressive and anxiety symptoms.

### Strengths and Limitations

Strengths of this study were the large sample size, the multiethnic population involved, and the use of internationally validated scales for assessing depressive symptoms and anxiety symptoms in economically underdeveloped areas. Moreover, the wide information collected included socioeconomic, lifestyles, physical and mental health status, other disorders, and chronic diseases. However, this study has some limitations. First, the study was cross-sectional in nature, so no causal inferences could be made. Second, the study population was enrolled voluntarily, and individuals who were suffering from severe depression or anxiety disorders were less likely to participate in this investigation, which might be another reason for the low prevalence. Third, due to the lack of information on cultural characteristics and differences in the urbanization process, the reasons for differences in depressive symptoms and anxiety symptoms among different multiple ethnic groups were not further explored. Fourth, we used the PHQ-2 and GAD-2 to measure depressive symptoms and anxiety symptoms. Although these two scales have been demonstrated to be suitable for application in community-based epidemically investigations and their reliability and validity were validated to be good for the present study population, overestimated or underestimated depressive symptoms and anxiety symptoms might still occur because of a lack of clinical professional psychiatric evaluations. Participants who were screened positive should be further evaluated with clinical diagnostic instruments or a direct interview with clinical psychologists to determine whether they met the criteria for both major depressive disorder and anxiety disorder.

## Conclusions

In this large population-based study of 93,078 adults from multiple ethnic groups in Southwest China, the prevalence of comorbid depressive and anxiety symptoms considerably varies among multiple ethnic groups. Stressful life events, physical and mental well-being and certain health conditions are positively associated with comorbid depressive and anxiety symptoms while the high frequency of physical activity and male alcohol consumption has negative associations. All the associations are stronger than depressive symptoms alone and anxiety symptoms alone. Since medical resources are relatively scarce in southwest multiethnic areas, these findings are considered valuable for policymakers to develop prevention programs in early identifying and intervening in the vulnerable populations with comorbid depressive and anxiety symptoms.

## Data Availability Statement

The original contributions presented in the study are included in the article/Supplementary Material, further inquiries can be directed to the corresponding authors.

## Ethics Statement

The studies involving human participants were reviewed and approved by Sichuan University Medical Ethical Review Board (K2016038). The patients/participants provided their written informed consent to participate in this study.

## Author Contributions

XZ, PL, and QL contributed to the study conception and design. QL and PW contributed to analyzed data and draft the first version of manuscript. YB, SW, ZS, and JZ contributed to collected and clear up data. HS, YL, and XL contributed to critically revised manuscript. All authors read and approved the final manuscript.

## Funding

This study was supported by the National Key R&D Program ‘Precision Medicine Initiative’ of China (Grants 2017YFC0907303 and 2017YFC0907302) and the Sichuan Science and Technology Program (Grants 2020JDJQ0014 and 2020YFS0216). The funding had no role in the design, analysis or writing of this manuscript.

## Conflict of Interest

The authors declare that the research was conducted in the absence of any commercial or financial relationships that could be construed as a potential conflict of interest.

## Publisher's Note

All claims expressed in this article are solely those of the authors and do not necessarily represent those of their affiliated organizations, or those of the publisher, the editors and the reviewers. Any product that may be evaluated in this article, or claim that may be made by its manufacturer, is not guaranteed or endorsed by the publisher.
