# Supplementary material for: Comorbid Depressive and Anxiety Symptoms and Their Correlates Among 93,078 Multiethnic Adults in Southwest China
Source: Front Public Health. 2021 Dec 14;9:783687. doi: 10.3389/fpubh.2021.783687 (PMC8712466; doi:10.3389/fpubh.2021.783687)
Supplement: Supplementary file 1 [file Data_Sheet_1.docx]

**SUPPLEMENTAL TABLE 1**│**Alcohol consumption in multiple ethnic groups and by gender by mental health status**

|  | Overall | Depression | Anxiety | Comorbid depressive and anxiety symptoms |
| --- | --- | --- | --- | --- |
|  | N=93078 | N=4947 (%) | N=5800 (%) | N=2721 (%) |
| **Han** |  |  |  |  |
| No | 28469 | 1454(5.1) | 1428(5.0) | 702(2.5) |
| Sometime | 18121 | 699(3.9) | 674(3.7) | 330(1.8) |
| Frequently | 8691 | 295(3.4) | 313(3.6) | 133(1.5) |
| **Dong** |  |  |  |  |
| No | 4113 | 486(11.8) | 684(16.6) | 317(7.7) |
| Sometime | 2242 | 215(9.6) | 275(12.3) | 123(5.5) |
| Frequently | 867 | 63(7.3) | 86(9.9) | 30(3.5) |
| **Bouyei** |  |  |  |  |
| No | 2917 | 233(8.0) | 363(12.4) | 141(4.8) |
| Sometime | 2277 | 134(5.9) | 211(9.3) | 79(3.5) |
| Frequently | 760 | 33(4.3) | 67(8.8) | 21(2.8) |
| **Yi** |  |  |  |  |
| No | 4436 | 317(7.2) | 429(9.7) | 208(4.7) |
| Sometime | 1175 | 59(5.0) | 97(8.3) | 42(3.6) |
| Frequently | 665 | 30(4.5) | 39(5.9) | 19(2.9) |
| **Miao** |  |  |  |  |
| No | 2495 | 323(12.9) | 427(17.1) | 227(9.1) |
| Sometime | 2371 | 242(10.2) | 297(12.5) | 143(6.0) |
| Frequently | 672 | 68(10.1) | 85(12.7) | 34(5.1) |
| **Bai** |  |  |  |  |
| No | 5063 | 146(2.9) | 157(3.1) | 89(1.8) |
| Sometime | 619 | 10(1.6) | 12(1.9) | 5(0.8) |
| Frequently | 424 | 7(1.7) | 8(1.9) | 3(0.7) |
| **Tibetan** |  |  |  |  |
| No | 4586 | 108(2.4) | 123(2.7) | 64(1.4) |
| Sometime | 1606 | 21(1.3) | 20(1.3) | 10(0.6) |
| Frequently | 509 | 4(0.8) | 5(1.0) | 1(0.2) |
| **Male** |  |  |  |  |
| No | 12318 | 634(5.2) | 641(5.2) | 332(2.7) |
| Sometime | 13893 | 542(3.9) | 580(4.2) | 265(1.9) |
| Frequently | 10982 | 393(3.6) | 439(4.0) | 175(1.6) |
| **Female** |  |  |  |  |
| No | 39761 | 2433(6.1) | 2970(7.5) | 1416(3.6) |
| Sometime | 14518 | 838(5.8) | 1006(6.9) | 467(3.2) |
| Frequently | 1606 | 107(6.7) | 164(10.2) | 66(4.1) |


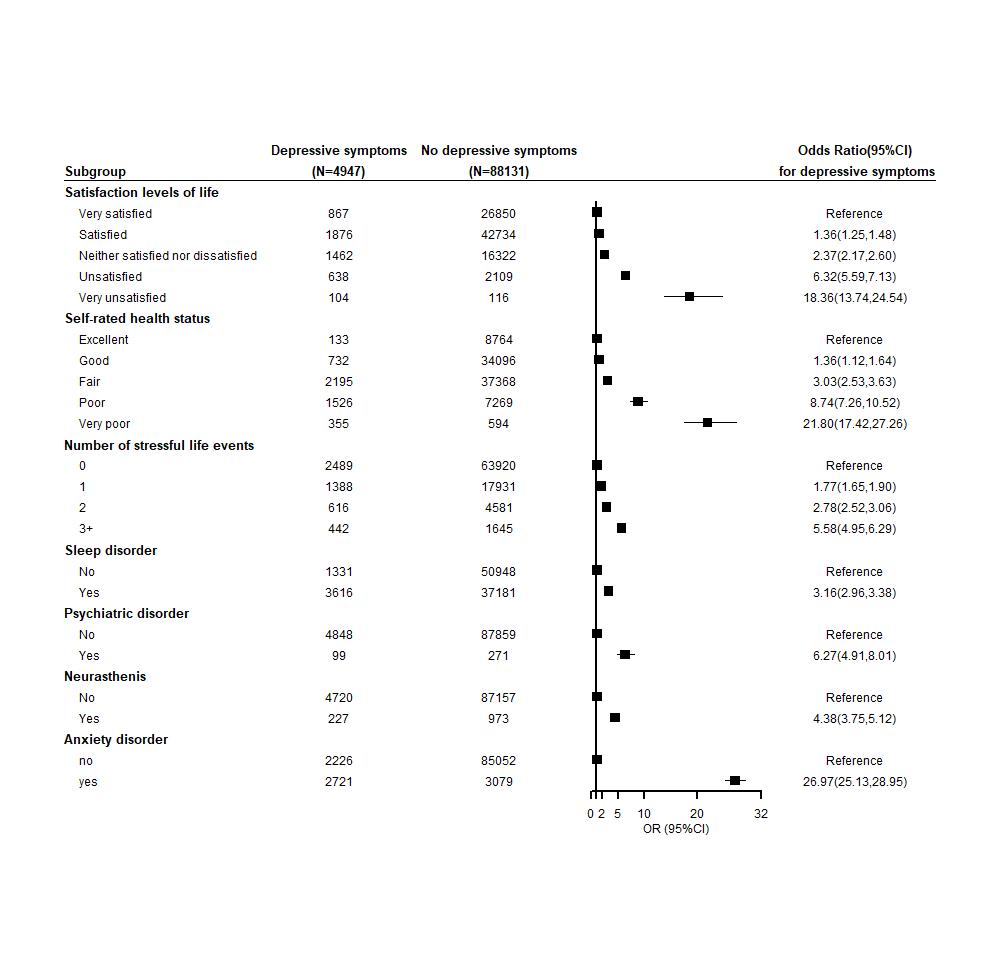


SUPPLEMENTAL FIGURE 1│Adjusted odds ratios by health-related conditions and status for depression. Each closed square represents an odds ratio, and the horizontal lines represent the 95% confidence interval (CI). All ORs were adjusted for age, sex, nationality, marriage, education level, occupation, income, smoking, alcohol consumption, tea consumption, BMI, physical activity and diseases.


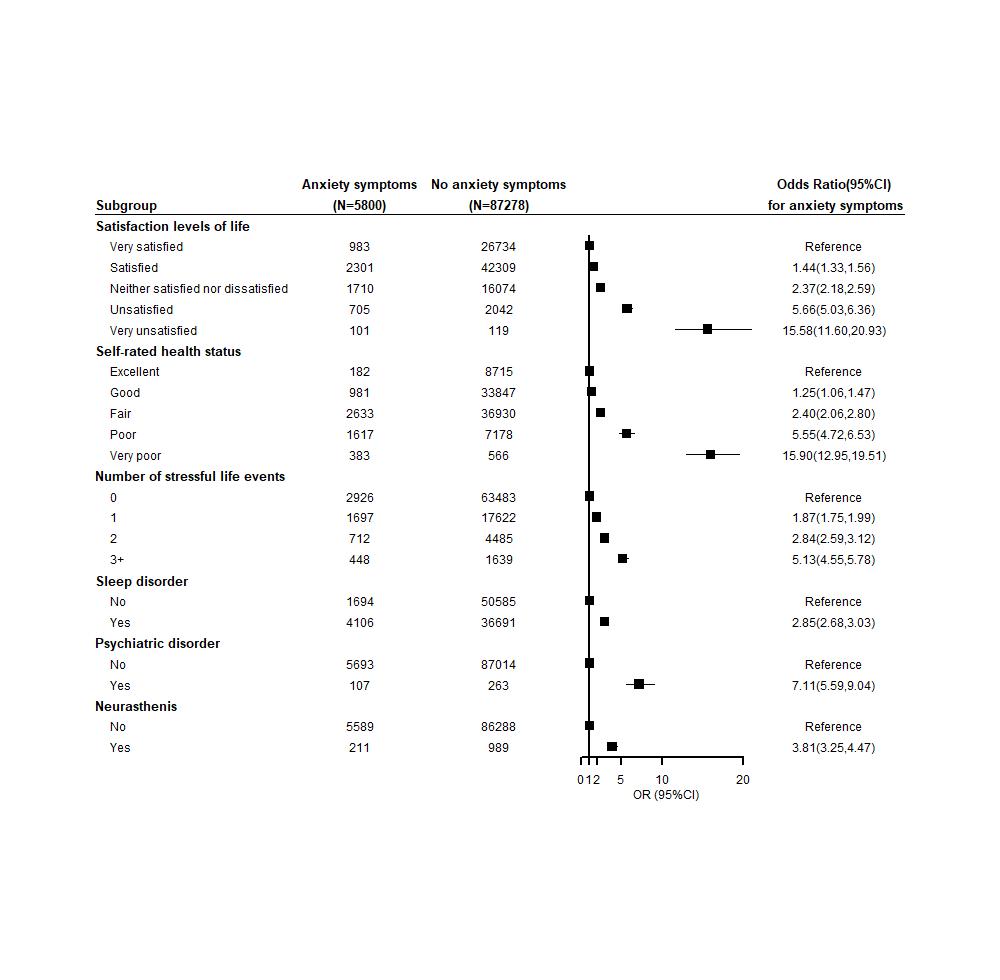


SUPPLEMENTAL FIGURE 2 │Adjusted odds ratios by health-related conditions and status for anxiety. Each closed square represents an odds ratio, and the horizontal lines represent the 95% confidence interval (CI). All odds ratios were adjusted for age, sex, nationality, marriage, education level, occupation, income, smoking, alcohol consumption, tea consumption, BMI, physical activity and diseases.
